# Response to Abudayeh and Fishchenko

**DOI:** 10.1097/PR9.0000000000001439

**Published:** 2026-05-19

**Authors:** Leslie R. Morse

**Affiliations:** Christine E. Lynn Rehabilitation Center, Department of Physical Medicine and Rehabilitation, University of Miami Miller School of Medicine, Miami, FL, USA

## *Letter to the Editor:*

We thank Drs. Abudayeh and Fishchenko for their thoughtful and constructive letter regarding our pilot randomized, double-blind, placebo-controlled trial of brivaracetam for spinal cord injury–related neuropathic pain.^1,6^ We appreciate their careful attention to methodological rigor and welcome the opportunity to clarify our rationale for allowing postrandomization changes in concurrent analgesics.

We agree that postrandomization escalation or modification of background pain medications represents an intercurrent event that may affect interpretation of treatment effects, particularly in small exploratory trials. In contemporary trial methodology, the ICH E9(R1) addendum emphasizes explicit definition of the estimand, that is, the precise treatment effect a study is designed to estimate, including how intercurrent events are handled.^3^ Within this framework, different strategies may appropriately address auxiliary medication changes depending on the scientific question being posed.

Our pilot trial was intentionally designed to estimate a treatment-policy (de facto) estimand, reflecting the effectiveness of brivaracetam in a real-world setting where rescue and concomitant analgesics remain available and may be adjusted by patients and their clinicians. Chronic pain trials commonly permit such auxiliary medications, and postrandomization adjustments are recognized as inherent to the fluctuating and refractory nature of neuropathic pain.^4,5^ A comprehensive review of 265 placebo-controlled trials in low back and neuropathic pain reported that 44% permitted rescue medication and nearly half allowed continuation of usual analgesics, underscoring that such designs are standard practice in chronic pain research.^4^ Similarly, high-quality evidence syntheses, including Cochrane reviews and large meta-analyses of neuropathic pain pharmacotherapy, have included trials that permitted rescue medication and stable background analgesics, including in spinal cord injury populations.^2,7^

Within the E9(R1) framework, an intention-to-treat analysis under these conditions estimates the combined effect of randomized treatment assignment and any clinically indicated background medication changes, often referred to as a de facto estimand.^3,5^ Importantly, prior methodological analyses note that when rescue medications are permitted, greater use in the placebo group may attenuate between-group differences and, therefore, yield conservative efficacy estimates rather than artificially inflate treatment effects.^4,5^ In this context, the greater need for analgesic escalation observed in the placebo group in our study is consistent with this established pattern.

Restricting or prohibiting concurrent analgesic modification may improve internal purity but does so at the cost of reduced generalizability and potentially ethical concerns in patients with severe, chronic pain.^5^ Particularly in a decentralized pilot study enrolling community-dwelling individuals with longstanding spinal cord injury, prohibiting clinically indicated medication adjustments over a 3-month period would not reflect routine clinical care and could increase attrition or patient burden. Our design, therefore, prioritized feasibility, safety, and preliminary signal detection under conditions that approximate real-world practice.

We agree that fully powered confirmatory trials should prospectively prespecify estimand strategies for auxiliary medication–related intercurrent events, incorporate stabilization periods where feasible, and include sensitivity analyses to explore robustness of results under alternative assumptions.^3,5^ However, in the setting of an exploratory pilot trial designed to assess feasibility and preliminary efficacy, allowance of clinically indicated changes in concurrent medications is consistent with established chronic pain methodology and with the ICH E9(R1) framework when targeting a treatment-policy estimand.

We appreciate the opportunity to clarify this aspect of the study design and are grateful for the authors' engagement with the methodological nuances of chronic pain trials.

## Disclosures

The author has no conflict of interest to declare.
